# Acetyl group for proper protection of β-sugar-amino acids used in SPPS

**DOI:** 10.1007/s00726-023-03278-1

**Published:** 2023-06-21

**Authors:** István Varga, Viktória Goldschmidt Gőz, István Pintér, Antal Csámpai, András Perczel

**Affiliations:** 1 Laboratory of Structural Chemistry and Biology, Institute of Chemistry, Eötvös Loránd University, Pázmány P. Stny. 1/A, Budapest, 1117 Hungary; 2 György Hevesy Doctoral School of Chemistry, Eötvös Loránd University, Budapest, Hungary; 3 ELKH-ELTE Protein Modeling Research Group, Pázmány P. Stny. 1/A, Budapest, 1117 Hungary; 4 Organic Chemistry Department, Eötvös Loránd University, Pázmány P. Stny. 1/A, Budapest, 1117 Hungary

**Keywords:** Sugar amino acids, Chimera/foldamer, Cleavage protocols, Solid-phase peptide synthesis

## Abstract

The synthesis of d-glucosamine-1-carboxylic acid based β-sugar amino acids (β-SAAs) is typically performed in nine consecutive steps via an inefficient OAc → Br → CN conversion protocol with low overall yield. Here, we present the improved and more efficient synthesis of both Fmoc-GlcAPC-OH and Fmoc-GlcAPC(Ac)-OH, β-SAAs consisting of only 4–5 synthetic steps. Their active ester and amide bond formation with glycine methyl ester (H-Gly-OMe) was completed and monitored by ^1^H NMR. The stability of the pyranoid OHs protecting the acetyl groups was investigated under three different Fmoc cleavage conditions and was found to be satisfactory even at high piperidine concentration (e.g. 40%). We designed a SPPS protocol using Fmoc-GlcAPC(Ac)-OH to produce model peptides Gly-β-SAA-Gly as well as Gly-β-SAA-β-SAA-Gly with high coupling efficiency. The products were deacetylated using the Zemplén method, which allows the hydrophilicity of a building block and/or chimera to be fine-tuned, even after the polypeptide chain has already been synthesized.

**Supplementary Information:**

The online version contains supplementary material available at 10.1007/s00726-023-03278-1.

## Introduction

Like polypeptides and proteins, oligosaccharides are evolutionarily fine-tuned polymers composed of a limited number of building blocks, and like polypeptides and proteins, they are involved in most cellular processes (Stern and Jedrzejas 2008; BeMiller 2008; Seeberger 2005; Roseman 2001; Robty 1998; Xiao et al. 2017; Varki 2022). Sugar amino acids (SAAs) (Risseeuw et al. 2013; Tian et al 2015; El Oualid 2004; Lohof et al. 1999, 2000; McDevitt and Lansbury 1996; von Roeden et al 1996; Goldschmidt Gőz et al. 2018; Nagy et al. 2017; Gruner et al. 2002a, b) combine several preferred properties of both amino acids and carbohydrates, and they also offer the possibility of conformational design from β-SAA-based foldamers (Gellman 1998) or chimeric building blocks, allowing the synthesis of stable helical or elongated biocompatible oligomers with predetermined polarity and spatial features. Due to their structural and stereochemical complexity, β-SAAs are versatile and tunable. For example, d-glucose (d-Glc) and d-mannose (d-Man) differ from each other only in the configuration of the C-2 atom, as C-2 epimers, yet they have markedly different conformational properties (Pigman and Horton 1972; Mayes et al. 2014; Rao 1998). Furthermore, structurally different foldamer building units (e.g. d-xylose (d-Xyl) and d-ribose (d-Rib), C-3 epimers (Nagy et al. 2017)) can generate different 3D folds and stabilize different secondary structural elements (Chandrasekhar et al. 2004; Gruner et al. 2002a, b; Menyhárd et al. 2017). While oligomers composed of -RibAFU(ip)- adopt a H12 helical structure, its C-3 epimer, namely the -XylAFU(ip)- building block, forms the broader H14 helix in solution.

SAAs with variable ring size (e.g. furanoid, pyranoid), configurational and conformational properties can be decorated with different functional groups, e.g. -OH, -NH_2_, -CH_2_-OH, -OAc, -NHAc, (Risseeuw et al. 2013; Gruner et al. 2002a, b; Tian et al. 2015; Gervay-Hague and Weathers 2002), than natural product SAAs such as sialic acids, neuraminic acid, etc. In line with the recently developed awareness and expectations, chemically synthesized SAAs are expected to retain beneficial properties of their natural counterparts, such as biocompatibility and biodegradability, as well as endogenous-like hydrophilicity or hydrophobicity. Typical examples include glucose derivatives (H-GlcAPC-OH), which yield d-glucosamine-based β-SAAs, and Fmoc- or Boc-protected derivatives (Fmoc-GlcAPC-OH, Fmoc-GlcAPC(Bn)-OH, Boc-GlcAPC(Ac)-OH (Suhara et al. 1996, 2002, 2006; von Roedern et al. 1996)).

**Scheme 1:**
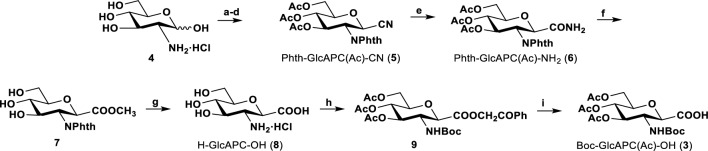
The stepwise synthesis of Boc-GlcAPC(Ac)-OH (**3**) β-SAA, using OAc → Br → CN chain elongation to form the carboxyl function. Conditions: **a-d**) ref. Suhara 1996, 46% overall yield, **e**) Ac_2_O, 30% HBr in AcOH, 24 h, rt, 72%; **e**) 30% HBr in AcOH, 3 h, rt, 85%; **f**) Dowex 50W-X8 [H^+^], MeOH, 16 h, 80 °C, 97%; **g**) *i)* 6 equiv. of LiOH, MeOH:H_2_O 3:1, 16 h 60 °C, *ii)* 3 M HCl, 3 h, reflux, 95% overall; **h**) *i)* 2 equiv of BOC-ON, Et_3_N, dioxane:H_2_O 1:1, 12 h, rt; *ii)* 2-bromoacetophenone, Et_3_N, DMF, 4 h, rt; *iii)* Ac_2_O, pyridine, 12 h, rt, 73% overall; **i**) H_2_, Pd/C, EtOAc:EtOH 2:1, 16 h, rt, 90%

The most efficient and widely used method for the synthesis of oligopeptides and chimeras is solid-phase peptide synthesis (SPPS), which has recently been adopted for β-SAAs. (Goldschmidt Gőz et al. 2019, 2021; Nagy et al. 2019; Farkas et al. 2021; Duong et al. 2021) However, both the active ester formation and the coupling reaction conditions require the protection of the nucleophilic -OH groups, similar to the case of Ser and Thr residues. Acetylation is one of the simplest and easiest ways of providing -OH protection in carbohydrate chemistry and has already been used in solution phase peptide synthesis by Suhara et al. (2006). They obtained the *N*-Boc and *O-*acetyl protected sugar amino acid (β-SAA) from d-glucosamine‧HCl after completing a nine-step reaction sequence (Scheme 1). From the monomer, they synthesized new dimer, trimer and tetramer ‘carbopeptoids’ using conventional solution phase peptide synthesis (Suhara et al. 2006). Since our intention was to use the β-SAA moiety in SPPS, we needed to thoroughly investigate the stability of the -OAc-protected β-SAAs under the conditions of the peptide coupling and Fmoc deprotection steps, as well as when the crude product was removed from the resin.

Although a large number of coupling reagents and active esters have been used in the past (Valeur and Bradley 2009; El-Faham and Albericio 2011), HOBt/DIC (Hydroxybenzotriazole/*N,N*-Diisopropylcarbodiimide) are now the most widely used reagents (Albericio et al. 1998). The PyBOP/DIEA (Benzotriazol-1-yloxytripyrrolidinophosphonium hexafluorophosphate/*N*,*N*-Diisopropylethylamine) reagent pair, which has been shown to minimize the risk of racemization, is also used to construct more challenging sequences. Recently, we have investigated and optimized different coupling reagents to generate GXXG model peptides (Goldschmidt Gőz et al. 2019; Nagy et al. 2019), where X was either a pyranoid (e.g. Fmoc-GlcAPU(Me)-OH) or a furanoid (e.g. Fmoc-RibAFU(ip)-OH) β-SAAs. To optimize both products formation and coupling as a function of time, their active ester formation was monitored by ^1^H NMR. For both furanoid and pyranoid β-SAAs, PyBOP/DIEA was found to be the best coupling reagent. Using this method, active esters are formed rapidly (within 20 min with high conversion: > 99%) and remain stable for at least 6–24 h, significantly longer than the duration of a typical coupling cycle (3 h).

Here, we demonstrate a shorter and scalable synthetic route for the preparation of the novel β-SAA coupling component Fmoc-GlcAPC-OH (**1**) and its per-*O*-acetylated derivative Fmoc-GlcAPC(Ac)-OH (**2**). Although *O*-acetyl protection is common in carbohydrate chemistry, and commonly used in the synthesis of glycopeptides and related compounds (Bermejo et al 2020), it is infrequently used in conventional SPPS. Therefore, in response to this synthetic challenge, we have fine-tuned protocols in which the reaction is monitored by NMR. Active ester formation and coupling with H-Gly-OMe was followed by time-resolved ^1^H NMR. Focusing on the different model peptides introduced earlier, both Ac-GXG-NH_2_ and Ac-GXXG-NH_2_ were prepared, where X represents the β-SAA. We show here, that the acetyl groups are highly stable under a variety of Fmoc and resin cleavage conditions, resulting in the fully protected oligopeptides. Furthermore, the *O*-acetyl groups can be subsequently removed by the standard Zemplén deacetylation method, widely used in carbohydrate chemistry, resulting in an unprotected and even more polar β-SAA-containing chimera.

## Results and discussion

### Synthesis of H-GlcAPC-OH derivatives

The synthesis of several H-GlcAPC-OH derivatives has already been described (Suhara et al. (loc cit; von Roeden et al. 1996), but the method published by Suhara et al. is the most widely accepted and used, leading to the *N*-Boc and *O*-Ac protected β-SAA: Boc-GlcAPC(Ac)-OH (**3**). However, we found that this route—which consists of 9 steps—is too long and that it could be shortened and optimized. Furthermore, we wanted to prepare building blocks suitable for SPPS (Goldschmidt Gőz et al. 2018; Nagy et al. 2017) and also for flow peptide chemistry (Farkas et al. 2021; Goldschmidt Gőz et al. 2021). Thus, in order to be compatible with Fmoc chemistry, the targeted β-SAA must be protected by Fmoc and Ac groups, respectively.

In the original Suhara synthesis, the key intermediate 3,4,6-tri-*O*-acetyl-2-deoxy-2-phthalimido-β-d-glucopyranosyl-1-carbonitrile (**5**) was obtained in four steps and the final product **3** was obtained in five consecutive steps. The chain elongation leading to the nitrile derivative (**5**), made by OAc → Br → CN exchange, are the key steps of this synthesis. Subsequently, **5** was hydrolyzed in three steps to give the fully unprotected d-glucosamine-1-carboxylic acid (H-GlcAPC-OH, **8**), which was then acetylated and *N*-protected by Boc to give Boc-GlcAPC(Ac)-OH (**3**), a molecule finally suitable for synthesis using solution-state protocols. This building block was used to complete the homooligomer synthesis with BOP/DIEA, a stepwise synthesis in solution, resulting in the dimer, trimer, etc. up to the hexamer (Suhara et al. 2006). The *O*-acetyl protection was removed after the final step using the Zemplén deacetylation method. Based on NMR and CD spectroscopy data, only the hexamer is long enough to adopt a 14-helix structure (Suhara et al. 2006). The -GlcAPC- building block can form oligomers by using different coupling agents (e.g. BOP/DIEA or HOBt/EDC‧HCl). However, the synthesis of the β-SAA building block is tedious in this way, and these synthons are only suitable for peptide synthesis in the solution state.

**Scheme 2:**
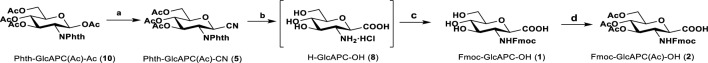
Improved synthesis of H-GlcAPC-OH based β-sugar amino acids (**1**, **2**). Reaction conditions: **a)** MeCN, TMSCN, BF_3_‧OEt_2_, rt, 18 h, 50%_,_ **b)** *i)* 12% NaOH in H_2_O, reflux (18 h), *ii)* 2 M HCl, reflux (18 h), **c)** Fmoc-OSu, MeOH, dioxane, rt (48 h) and **d)** Ac_2_O, Pyridine, rt (18 h)

On the other hand, our fine-tuned procedure is based on *N*-Fmoc and *O*-Ac protected β-SAA suitable for SPPS. Starting from the commercially available 1,3,4,6-tetra-*O*-acetyl-2-deoxy-2-phthalimido-d-glucopyranose, Phth-GlcAPC(Ac)-Ac (**10**), chain elongation with TMSCN led directly to the nitrile derivative **5** (Scheme 2) (Myers and Lee 1984).The hydrolysis of the nitrile (**5**) gave the HCl salt of the unprotected H-GlcAPC-OH (**8**) in two steps: (a) Zemplén deacetylation removed the acetyl protection; (b) hydrolysis with 12% aqueous NaOH was carried out to partially remove the *N*-Phth protection and hydrolysis with 2 M HCl gave H-GlcAPC-OH (**8**). Then, this was converted to the Fmoc protected derivative, Fmoc-GlcAPC-OH (**1**). In the final step, the OH groups were conventionally acetylated (Ac_2_O in pyridine) to give the fully protected β-SAA, Fmoc-GlcAPC(Ac)-OH (**2**), which was shown (as detailed below) to be suitable for SPPS.

Our method is more efficient than that of Suhara et al. who obtain their final compound, Boc-GlcAPC(Ac)-OH, in 9 steps with an overall yield of 26%. We achieved our target molecules Fmoc-GlcAPC-OH (**1**) and obtained Fmoc-GlcAPC(Ac)-OH (**2**) in four and five steps, with overall yields of 36% and 29%, respectively. The process is also more environmentally friendly by reducing the number of synthetic steps. The key step, chain elongation, is performed in a single step. After their formation, both **1** and **2** can be precipitated with 2 M HCl. Molecules **5** and **2** were both successfully recrystallized from ethanol, eliminating column chromatography and providing a greener synthetic route.

### Active ester formation

**Fig. 1:**
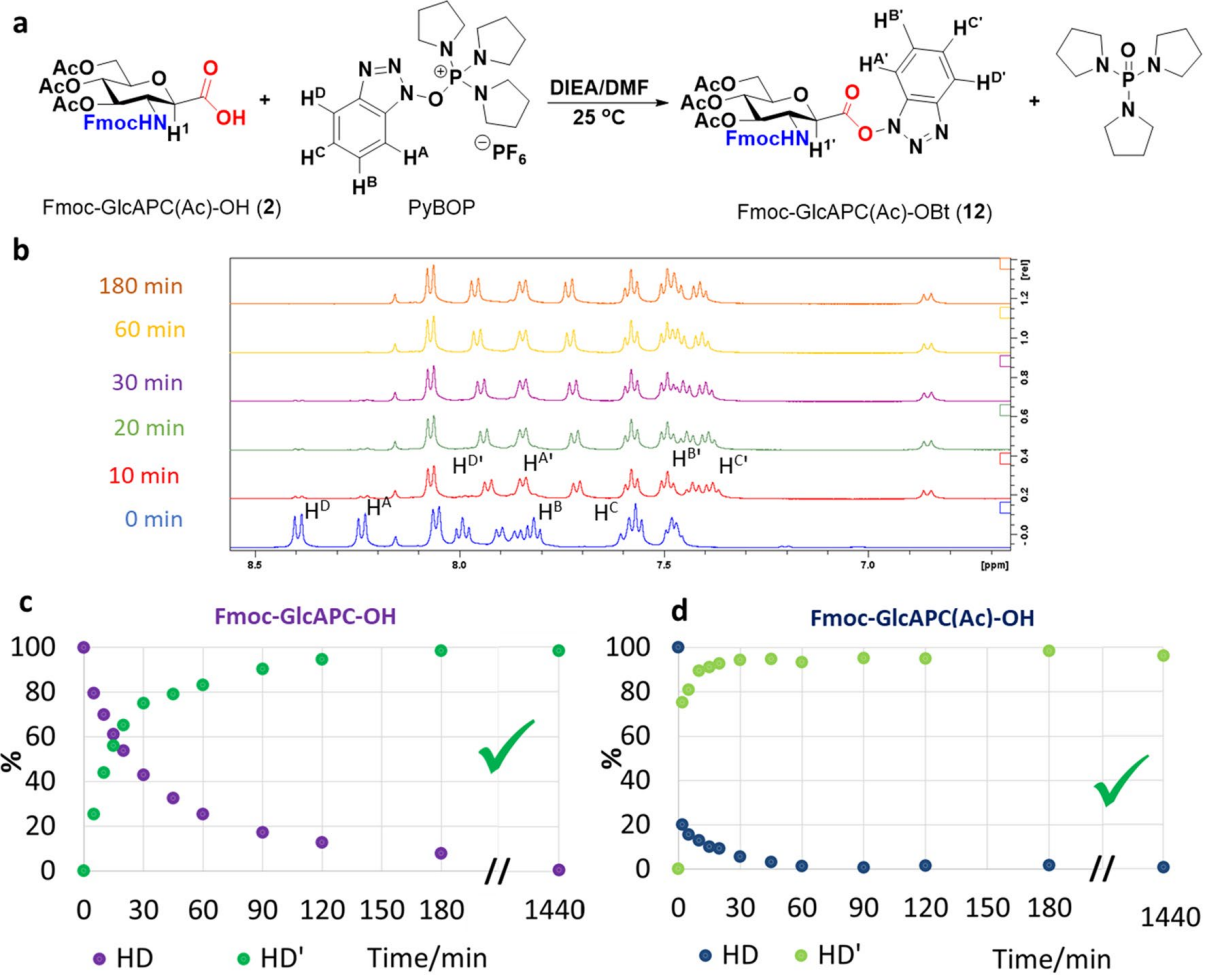
Monitoring of active ester formation using ^1^H NMR spectroscopy. Both Fmoc-GlcAPC-OH (**1**) and Fmoc-GlcAPC(Ac)-OH (**2**) form the corresponding active esters, which then, retain their stability even after 24 h. **a)** The reaction of Fmoc-GlcAPC(Ac)-OH (**2**) with PyBOP/DIEA in DMF-d_7_ gives the active ester **12**. **b)** The active ester formation was monitored by time-resolved ^1^H NMR spectra: chemical shift changes of selected aromatic protons were monitored. **c)** Using the integrals of the H^D^ (8.24 ppm) and H^D’^ (7.74 ppm) resonances, the active ester formation of Fmoc-GlcAPC-OH (**1**) was quantitatively determined as a function of time. **d)** Using the integrals of the H^D^ (8.27 ppm) and H^D’^ (7.83 ppm) resonances, the active ester formation of Fmoc-GlcAPC(Ac)-OH (**2**) was quantitatively determined as a function of time

We have previously investigated several coupling reagents and conditions to obtain the best method for β-SAAs coupling (Nagy et al. 2019). Before proceeding with SPPS, we performed ^1^H NMR measurements to determine if the PyBOP/DIEA coupling reagent pair was suitable and to determine the optimal coupling conditions. We monitored the active ester formation of the β-SAAs (Fmoc-GlcAPC-OH and Fmoc-GlcAPC(Ac)-OH) as a function of time (Goldschmidt Gőz et al. 2019; Nagy et al. 2019). In DMF (Fig. 1a), the hydroxybenztriazol moiety of PyBOP transforms into the active ester moiety of both Fmoc-GlcAPC-OBt (**11**) and Fmoc-GlcAPC(Ac)-OBt (**12**), whose formation was indicated by the characteristic chemical shift changes of the aromatic H^D^ with respect to the parent H^D’^ (Fig. 1b). The formation of the active ester was monitored for both β-SAA derivatives and we found that the formation of Fmoc-GlcAPC-OBt (**11**) requires ~ 1 h, resulting in an almost quantitative (96%) conversion (Fig. 1c and SFigure 1). We have also found that the active ester remains stable in solution for up to 24 h at room temperature. The reaction with Fmoc-GlcAPC(Ac)-OH (**2**) was slower (~ 3 h), but again the conversion was almost quantitative (~ 99%) and the product was stable even after 24 h. Note how useful the NMR monitoring of the reaction is for these β-SAAs, we were able to determine that the activation is slower than expected (1–3 h), but in return the active ester formations are almost quantitative.

### Coupling with the active esters

**Fig. 2:**
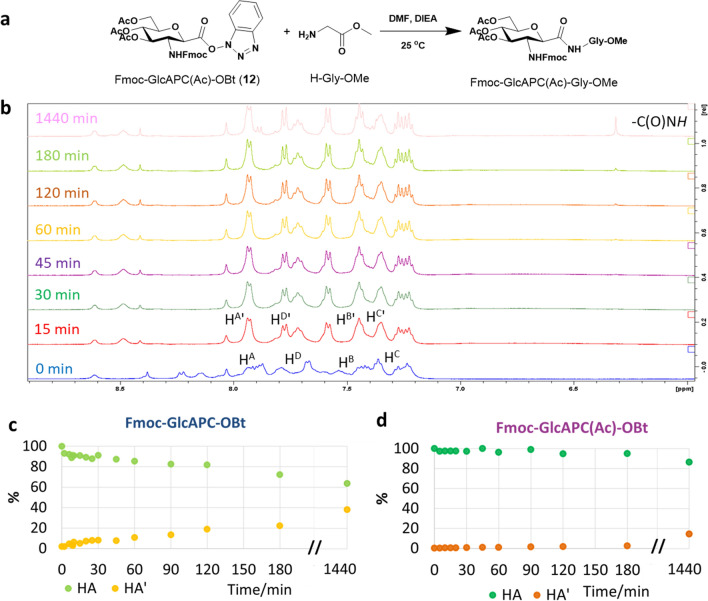
Monitoring the peptide bond formation of two β-SAA active esters with H-Gly-OMe, by using ^1^H NMR spectroscopy. Both Fmoc-GlcAPC-OBt (**11**) and Fmoc-GlcAPC(Ac)-OBt (**12**) form the corresponding peptide bond, Fmoc-β-SAA-Gly-OMe, but slowly. **a)** The coupling reaction scheme of Fmoc-GlcAPC(Ac)-OBt (**12**) with H-Gly-OMe in DMF-d_7_. **b**) The peptide bond formation monitored by ^1^H NMR spectra as a function of the time, via chemical shift changes of selected aromatic resonances. **c**) Using the integrals of the H^A^ (7.86 ppm) and H^A’^ (8.01 ppm) resonances, the peptide bond formation with Fmoc-GlcAPC-OBt (**11**) was quantitatively determined (in %) as function of the time. **d**) Using the integrals of the H^D^ (7.79 ppm) and H^D’^ (7.77 ppm) resonances, the peptide bond formation with Fmoc-GlcAPC(Ac)-OBt (**12**) was quantitatively determined (in %) as a function of the time

The coupling of the two active β-SAAs esters, Fmoc-GlcAPC-OBt (**11**) and Fmoc-GlcAPC(Ac)-OBt (**12**), was investigated with glycine, the simplest and most flexible proteinogenic amino acid residue. The C-terminal of Gly was protected as a methyl ester: H-Gly-OMe. Slow amide bond formation was observed for both Fmoc-GlcAPC-OBt (**11**) and Fmoc-GlcAPC(Ac)-OBt (**12**) in the presence of 2 equivalents of H-Gly-OMe and 6 equivalents of DIEA: even after 24 h the conversion was only 63% and 17%, respectively. (Fig. 2 and SFigure 2). Although this pilot reaction was too slow and the conversion was insufficient for direct implementation for SPPS, it should also be remembered that these initial experiments were carried out in an NMR tube without any stirring. In the following, we will show that the same coupling reaction proceeds more rapidly and more effectively when the reaction conditions are optimized for SPPS.

### Stability of the acetyl groups in chimera containing the -GlcAPC(Ac)- subunit

**Fig. 3:**
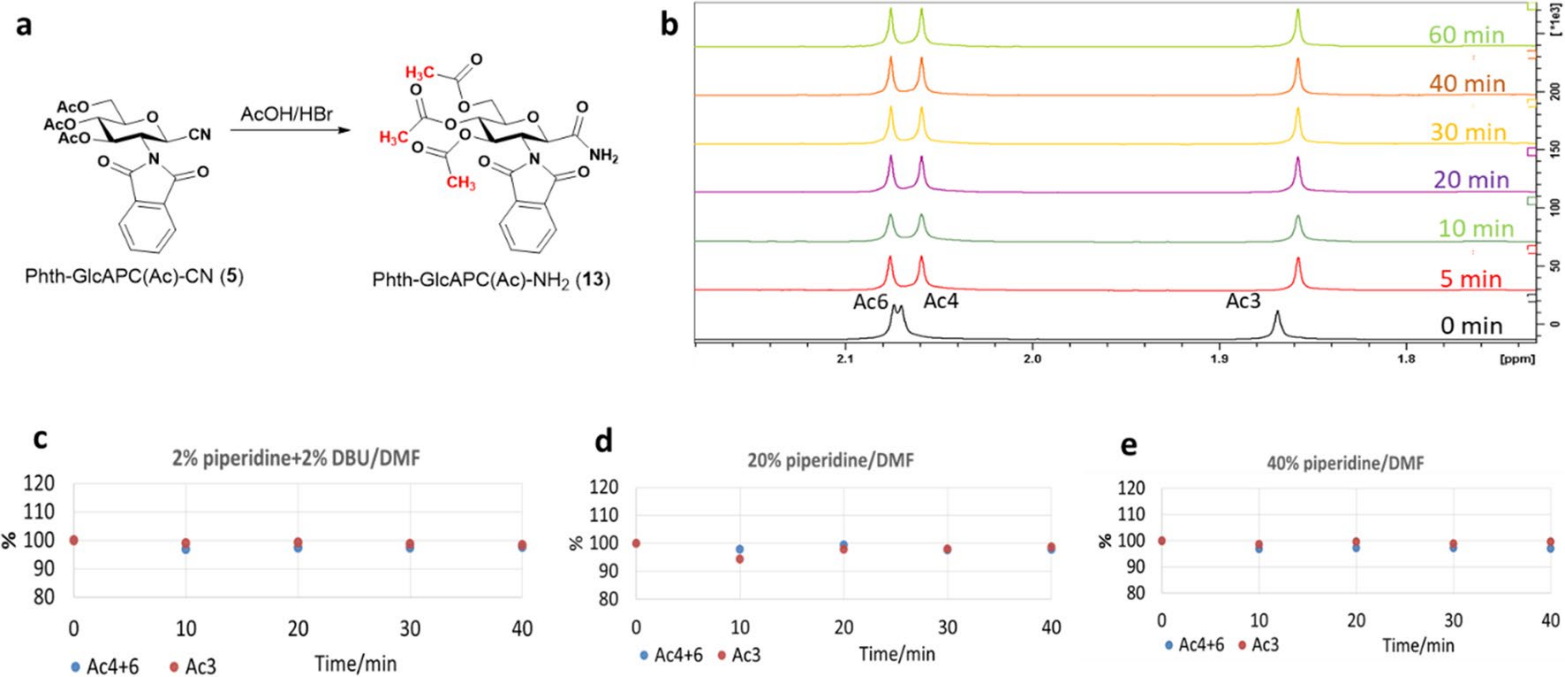
Investigation of the stability of *O*-acetyl groups under the different Fmoc cleavage conditions: stabilities appear to be preserved for 40 min. **a**) The synthesis scheme of a model amide derivative, Phth-GlcAPC(Ac)-NH_2_ (**13**). **b**) The stability of *O*-acetyl groups at 25 °C monitored by ^1^H NMR as function of the time, via chemical shift changes of selected *O*-acetyl protons: **c**) **2**% piperidine and 2% DBU in DMF-d_7_, **d**) **20**% piperidine in DMF-d_7_ and **e**) **40**% piperidine in DMF-d_7_

As our intention was to develop Fmoc chemistry suitable β-SAAs using acetylated derivatives, e.g. Fmoc-GlcAPC(Ac)-Gly-OMe, the question arises to what extent an acetyl protecting group resists cleavage conditions. Therefore, we investigated the stability of this group under conventional Fmoc cleavage conditions and beyond. The *N*-phthaloyl-protected model compound Phth-GlcAPC(Ac)-NH_2_ (**13**) was used, as the Phth-group is an inexpensive and simple amide protecting group (Fig. 3). Acetyl groups were found to be stable under conventionally accepted Fmoc cleavage conditions at room temperature (rt), such as 20% piperidine in DMF, or its equivalent 2% piperidine with 2% DBU in DMF (Fig. 3c–d and SFigure 3–5). Furthermore, even under the harshest conditions of flow chemistry (Farkas et al. 2021), 40% piperidine in DMF, the *O*-Ac group remains intact at 25 °C. In conclusion, acetyl groups seem to be suitable to protect -OH groups of β-SAAs moieties in SPPS, at least if (i) the chimera synthesis is not too long, (ii) the sequence is not too long and (iii) the sugar amino acid is not too close to the C-terminus of the sequence.

#### Coupling on solid phase

Our end products (**1**,** 2**) are promising candidates for coupling to SPPS. Having both the *O*-acetylated Fmoc-GlcAPC(Ac)-OH (**2**) and its “free” derivative Fmoc-GlcAPC-OH (**1**) in hand, SPPS was investigated for both. Note that the free β-SAA **1** has a total of 3 free -OH groups as nucleophiles, which could cause miscoupling, especially that of the primer -OH of the C-6 position. Armed with the previously established NMR data on active ester formation and coupling data, SPPS was used to generate the Ac-GXXG-NH_2_ model chimera (X = β-SAA: -GlcAPC- and -GlcAPC(Ac)-). Using this model peptide, we investigated all 3 different conditions of amide bond formation, namely those of α-β, β-β and β-α amide formation. For Fmoc-Gly-OH coupling, DIC/HOBt was used (1 h coupling time), whereas for β-SAAs PyBOP/DIEA was used (3 h). For Fmoc cleavage, 2% piperidine and 2% DBU in DMF protocol was used. When using Fmoc-GlcAPC-OH, a building block with free -OH groups, we found low coupling efficiencies (5–25% in Table 1). Furthermore, raw material analysis showed that the desired model compound was not obtained due to the large number of free -OH groups, e.g. MS data (see SFigure 11 and 16).

**Table 1:** Selected experimental data of the SPPS of chimera Ac-GXXG-NH_2_, by using **Fmoc-GlcAPC-OH** and **Fmoc-GlcAPC(Ac)-OH** β-sugar amino acids

SPPS with -GlcAPC- base β-SAAs		Tentagel^®^ S RAM resin		
		160 mg	50 mg	150 mg
Resin	Coupling with	**Fmoc-Gly-OH**^**a**^		
	Resin capacity (mmol/g)	0.21	0.23	0.23
	Coupling time (h)	1	1	1
Fmoc-G-Resin	Coupling with	**Fmoc-GlcAPC-OH**^**b**^	**Fmoc-GlcAPC(Ac)-OH**^**b**^	
	Coupling time (h)	3	3	3
	Resin capacity (mmol/g)	0.19	0.21	–
	Efficacy of coupling (%)	90	91	–
Fmoc-XG-Resin	Coupling with	**Fmoc-GlcAPC-OH**^**b**^	**Fmoc-GlcAPC(Ac)-OH**^**b**^	
	Coupling time (h)	3	3	3
	Resin capacity (mmol/g)	< 1%^c,d^	0.19	0.21
	Efficacy of coupling (%)	< 5%	90	91
Fmoc-XXG-Resin	Coupling with	**Fmoc-Gly-OH**^**a**^		
	Coupling time (h)	1	1	1
	Resin capacity (mmol/g)	0.053	0.16	0.19
	Efficacy of coupling (%)	28	84	90
Cleaving cocktail in %		TFA:DCM:TIS:H_2_O 95:2.5:2.5	TFA:DCM:TIS:H_2_O 50:45:2.5:2.5	TFA:TIS:H_2_O 95:2.5:2.5
Ac-GXXG-NH_2_	Overall yield for crude product (mg, %)	24 mg, > 95%	4 mg, 69%	25 mg, 90%
Results base on analytical data (MS)		**No product**	**Per-*O*-acetyl**	**Per-*O*-acetyl**

^a^Coupling agents were DIC/HOBt, coupling time was 1 h

^b^Coupling agents were PyBOP/DIEA, coupling time was 3 h

^c^Resin capacity was too low, did not measured

^d^After double coupling

*Fmoc-Gly-OH* *N*-(9-Fluorenylmethoxycarbonyl) glycine

On the other hand, the per-*O*-acetylated Fmoc-GlcAPC(Ac)-OH was successfully incorporated into the Ac-GXXG-NH_2_ model chimera: both coupling efficiencies (84–91%) and yields (69–90%) are acceptable (Table 1). We have previously shown that all protecting groups used for Fmoc/tBu SPPS protocols can be successfully removed during the final cleavage step using an acid-reduced cleavage mixture containing 50% TFA (Goldschmidt Gőz et al. 2021; Duong et al. 2021). Using the same mixture in this case, we found that the crude model chimera Ac-GXXG-NH_2_ was isolated with all *O*-acetyl groups successfully retained. Furthermore, even when using the 95% TFA mixture, the *O*-acetyl protecting groups remain intact as confirmed by MS (SFigure 15). Thus, both the milder and the harsher TFA conditions allow to obtain the desired peracetylated chimera.

#### Deacetylation

To further investigate the stability of the *O*-acetyl protecting groups, Ac-GXG-NH_2_ was synthesized and studied, as partial deacetylation is easy to detect on this simple chimera (SFigure 9). The final cleavage was carried out at 40 °C with 95% TFA and we found that the fully *O*-acetylated chimera is intact. Neither the MS spectrum nor the HPLC chromatogram show any sign of decomposition (SFigure 10 and 14).

To obtain the fully unprotected chimera, the Zemplén deacetylation method, a reaction widely used in carbohydrate chemistry to remove acetyl protection, was performed on Ac-GXG-NH_2_. As the deacetylation was performed in MeOH, which induces the collapse of the S RAM Tentagel^®^ resin, the reaction was performed after the final cleavage. The purified per-*O*-acetyl Ac-GXG-NH_2_ was dissolved in MeOH and a few drops of 2 M NaOMe/MeOH solution were added. After 1 h at room temperature, the reaction was stopped by adding TFA to the mixture. The result was a fully -OH deprotected Ac-GXG-NH_2_ chimeric peptide (SFigure 13 and 17). In summary, the *O*-acetyl groups were successfully removed by the Zemplén deacetylation method, whereas the conventional 50% or 95% TFA final cleavage mixture did not interfere with the *O-*acetyl groups.

## Conclusion

In the present work, new β-sugar amino acids, namely Fmoc-GlcAPC-OH (**1**) and Fmoc-GlcAPC(Ac)-OH (**2**), suitable for SPPS were synthesized in only four and five synthetic steps. Using NMR measurements, we found that the corresponding active esters are formed with PyBOP/DIEA in 1–3 h and when coupled with an α-amino acid, H-Gly-OMe, they form a β-α-amide bond. The stability of the *O*-acetyl protecting groups was confirmed by ^1^H NMR under Fmoc cleavage conditions using Phth-GlcAPC(Ac)-NH_2_ (**13**) as a model. Both Ac-GXG-NH_2_ and Ac-GXXG-NH_2_ chimeras with *O*-acetyl protected β-SAA (**2**) were successfully synthesized in SPPS: both coupling efficiencies and overall yields were found to be sufficient. The acetyl groups were removed by the Zemplén deacetylation method after final resin cleavage. In conclusion, it is possible to prepare chimeric peptides containing β-α-, α-β- and/or β-β-amide linkages using the new and efficiently synthesized β-SAA, Fmoc-GlcAPC(Ac)-OH (**2**). In addition, this biocompatible synthetic product is even more versatile and tunable, because the O-Ac groups of the β-SAA can be retained or removed at the end of the synthesis.

## Experimental

### General information

Analytical data for all compounds (HPLC chromatograms, NMR and MS spectra) can be found in Supporting information, in the online version.

Reagents, materials and solvents were purchased from Sigma-Aldrich, Merck, Reanal, VWR and Iris Biotech. Moisture-sensitive solvents were dried on molecular sieve (3 Å), while acetonitrile was distilled from CaH_2_. Solvents and reagents for MS measurements were purchased from VWR. TLC was performed on silica gel 60 F254, 230 mesh (E. Merck) and spots were detected by UV light (254 nm), charring with 5% H_2_SO_4_ solution.

Chimera peptides were measured by RP-HPLC on Aeris™ 3.6 μm peptide XB-C18 100 Å, LC Column 250 × 4.6 mm with eluents 0.1% TFA in H_2_O (A) and 0.08% TFA, 95% ACN/5% H_2_O (B), flow rate 0.9 ml/min and UV detection at 220 and 280 nm. Gradient was as follow: 0 min: 5% B, 30 min: 95% B, 33 min: 95% B, 33.1 min: 5% B, 45 min: 5% B.

NMR experiments were performed at 298 K on Bruker Avance DRX 500 MHz spectrometer equipped with TXI probe with z-gradient, operating at 500.13 MHz for ^1^H and 125.76 MHz for ^13^C. The sample concentrations ranged from 0.1 M to 0.2 M. Spectra were recorded in DMF-d_7_ using the solvent residual peak as the ^1^H internal reference. Spectra evaluation was done with TopSpin 4.1.1 software.

The mass spectrometry measurements were performed using a Bruker Amazon SL ion trap mass spectrometer equipped with an electrospray ion source.

**3,4,6-Tri-*O*-acetyl-2-deoxy-2-phthalimido-β-d-glucopyranosyl-1-carboxamide (Phth-GlcAPC(Ac)-CN, 5) **Tetra-*O*-acetyl-2-deoxy-2-phthalimido-d-glucopyranose (3.68 g, 7.7 mmol, **10**) was dissolved in anhydrous acetonitrile (25 ml). Then, TMSCN (1.5 ml, 1.5 equiv) and BF_3_‧OEt_2_ (1.5 ml, 1.6 equiv) were added to this solution. The reaction mixture was stirred at room temperature overnight. After TLC (toluene:EtOAc 3:2) indicated the completion of the reaction, water (10 ml) was added to the reaction mixture, followed by 30 min of stirring. The solution was concentrated in *vacuo*. Then, CHCl_3_ (40 ml) was added to the residue orange oil, and the solution was washed three times with water. The organic phase was dried (MgSO_4_), filtered and concentrated in *vacuo*. The remaining crude product was crystallized from EtOH, twice. Yield: 1.55 g (40%). TLC (toluene:EtOAc, 3:2 v/v): *R*_f_ = 0.51; [α]_D_^22^ =  + 65.3 (c = 1.06, CHCl_3_), lit. (Myers 1984) [α]_D_^22^ = 68.8 (c = 1.03, CHCl_3_)^1^H NMR (250 MHz, CDCl_3_) *δ* 7.89–7.74 (m, 4H, Phth-H), 5.72 (t, ^3^*J*_H.H_ = 9.73 Hz, 1H, H-3), 5.35 (d, ^3^*J*_H.H_ = 10.83 Hz, 1H, H-1), 5.17 (t, ^3^*J*_H.H_ = 9.66 Hz, 1H, H-4), 4.64 (t, ^3^*J*_H.H_ = 10.58 Hz, 1H, H-2), 4.31–4.13 (m, 2H, H-6), 3.89–3.83 (m, 1H, H-5).

**2-Deoxy-2-(9-fluorenylmethoxycarbonylamino)-β-d-glucopyranosyl-1-carboxylic acid (Fmoc-GlcAPC-OH, 1)** Phth-GlcAPC(Ac)-CN (**5**, 1 g, 2.25 mmol) was suspended in 12 m/m% NaOH solution (40 ml) and refluxed at 18 h. Then, *cc* HCl was added into the solution to get 2 M HCl concentration, and it was continued boiling at 18 h. After TLC (MeOH:AcOH 6:1) showed completion of the reaction, the mixture was left to cool to room temperature and filtered. The remaining solution was extracted with EtOAc (three times). Then, the water phase was concentrated in *vacuo* to remove HCl gas, and the remaining solution was lyophilized. Then, the dried product was dissolved in water/methanol (2/1, 15 ml). The pH of the solution was set to pH 8 with saturated aqueous NaHCO_3_ solution. Then, a solution of Fmoc-OSu (1.8 g, 1.1 equiv) in THF (15 ml) was added, and the pH was again set to pH 8 with NaHCO_3_ solution. The reaction mixture was stirred at room temperature for 2 days. After the reaction was completed, it was concentrated in *vacuo* to remove organic solvents. The remaining aqueous solution was diluted to 15 ml with water, pH set to pH 8 and extracted with EtOAc (5 times). The pH of the aqueous phase was set to 2 and it was cooled to 0 °C. The precipitate was filtered and dried. Yield: 0.85 g (88%). TLC (EtOAc:AcOH:H_2_O, 8:2:1 v/v/v): *R*_f_ = 0.34; [α]_D_^22^ =  + 19.8 (c = 0.05, DMF), ^1^H NMR (500 MHz, CDCl_3_) *δ* 7.99 (d, ^3^*J*_H,H_ = 6.4 Hz, 2H, Fmoc-H_D_); 7.88 (t, ^3^*J*_H,H_ = 8.5 Hz, 2H, Fmoc-H_A_); 7.44 (td, ^3^*J*_H,H_ = 14.8 and 2.2 Hz, 2H, Fmoc-H_C_); 7.30 (td, ^3^*J*_H,H_ = 7.3 and 2.2 Hz, 2H, Fmoc-H_B_); 4.21 (dd, ^3^*J*_H,H_ = 16.2 and 2.7 Hz, 1H, Fmoc CH_1_); 4.21 (t, ^3^*J*_H,H_ = 14.5 Hz, 1H, Fmoc CH_2A_); 4.15 (dd, ^3^*J*_H,H_ = 16.7 and 2.7 Hz, 1H, Fmoc CH_2B_); 3.95 (d, ^3^*J*_H,H_ = 10.2 Hz, 1H, H-1); 3.82 (m, 1H, H-6_A,B_); 3.72 (overlapped td, ^3^*J*_H,H_ = 10.2 and 4.3 Hz, 1H, H-2); 3.56 (m, 1H, H-5); 3.36 (dd, ^3^*J*_H,H_ = 4.5 and 3.2 Hz, 1H, H-4); 3.28 (dd, ^3^*J*_H,H_ = 4.8 and 8.6 Hz, 1H, H-3) ^13^C NMR (125 MHz, CDCl_3_) *δ* 170.7 (COOH); 156.3 (NH*C*(O)); 144.4 (Fmoc Ar_2_-C1); 144.2 (Fmoc Ar_1_-C1); 141.2 (Fmoc Ar_2_-C6); 141.1 (Fmoc Ar_1_-C6); 128.07 (Fmoc Ar_1_-C4); 128.04 (Fmoc Ar_2_-C4); 127.6 (Fmoc Ar_2_-C3); 127.5 (Fmoc Ar_1_-C3); 125.9 (Fmoc Ar_1_-C2); 125.7 (Fmoc Ar_2_-C2); 120.54 (Fmoc Ar_1_-C5); 120.5 (Fmoc Ar_2_-C5); 81.5 (C1); 78.3 (C5); 75.1 (C3); 70.9 (C4); 66.03 (Fmoc CH_2_); 61.5 (C6); 55.3 (C2); 47.1 (Fmoc C(2Ar)).

**3,4,6-Tri-*O*-acetyl-2-deoxy-2-(9-fluorenylmethoxycarbonylamino)-β-d-glucopyranosyl-1-carboxylic acid (Fmoc-GlcAPC(Ac)-OH, 2)** Fmoc-GlcAPC-OH (**1**, 1 g, 1.86 mmol) was suspended in pyridine (5 ml) and cooled to 0 °C, then, Ac_2_O (3 ml) was slowly added to this solution, and it was left to warm to room temperature, and stirred overnight. After TLC (EtOAc:MeOH 4:1) shows completion of the reaction, the reaction mixture was poured to ice-water (25 ml). The product was precipitated and filtered. The crude product was recrystallized from EtOH. Yield: 1.1 g (85%). TLC (MeOH:EtOAc:AcOH, 9:1:0.1 v/v/v): *R*_f_ = 0.31; [α]_D_^22^ =  + 193.5 (c = 0.047, DMF), ^1^H NMR (500 MHz, CDCl_3_) *δ* 7.89 (d, ^3^*J*_H,H_ = 7.6 Hz, 2H, Fmoc-H_D_); 7.64 (dd, ^3^*J*_H,H_ = 11.1 and 7.8 Hz, 2H, Fmoc-H_A_); 7.41 (t, ^3^*J*_H,H_ = 6.8 Hz, 2H, Fmoc-H_C_); 7.31 (t, ^3^*J*_H,H_ = 7.1 Hz, 2H, Fmoc-H_B_); 5.37 (t, ^3^*J*_H,H_ = 10.4 Hz, 1H, Fmoc CH_2A_); 4.98 (t, ^3^*J*_H,H_ = 9.6 Hz, 1H, Fmoc CH_2B_); 4.35 (d, ^3^*J*_H,H_ = 11.3 Hz, 1H, H-1); 4.23 (overlapped m, 1H, Fmoc CH_1_); 4.21 (m, 1H, H-6_A_); 4.19 (m, 1H, H-6_B_); 4.16 (m, 1H, H-5); 4.11 (dd, ^3^*J*_H,H_ = 10.3 and 1.2 Hz, 1H, H-3); 3.98 (dd, ^3^*J*_H,H_ = 10.3 and 10.1 Hz, 1H, H-2); 3.93 (m, 1H, H-5); 2.0 and 1.9 (s, 9H, Ac-CH_3_) ^13^C NMR (125 MHz, CDCl_3_) *δ* 170.2 (COOH); 169.9, 169.6, 169.2 (Ac-*C*(O)) 156.1 (NH*C*(O)); 144.4 (Fmoc Ar_2_-C1); 144.0 (Fmoc Ar_1_-C1); 141.2 (Fmoc Ar_2_-C6); 141.1 (Fmoc Ar_1_-C6); 127.7 (Fmoc Ar_1_-C4); 127.7 (Fmoc Ar_2_-C4); 127.2 (Fmoc Ar_1,2_-C3); 125.5 (Fmoc Ar_1_-C2); 125.4 (Fmoc Ar_2_-C2); 120.1 (Fmoc Ar_1,2_-C5); 77.4 (C1); 75.5 (C5); 73.7 (C3); 68.9 (C4); 66.5 (Fmoc CH_2_); 62.4 (C6); 53.6 (C2); 47.0 (Fmoc C(2Ar)).

### Peptide synthesis

For the SPPS S RAM Tentagel^®^ resin was used (nominal capacity 0.24 mmol/g). Resin was swollen in DCM. The first step was the removal of the Fmoc group with common method (2% piperidine and 2% DBU in DMF, 3 + 17 min). The successful cleavage was analyzed by the Kaiser test. After that, the coupling of αAAs to resin was made by reagent pairs HOBt/DIC (αAA:HOBt:DIC 1:1:1) in DMF for 1 h, while that of βSAA either Fmoc-GlcAPC-OH (**1**) or Fmoc-GlcAPC(Ac)-OH (**2**) was accomplished by PyBOP/DIEA (βSAA:PyBOP:DIEA 1:1:2) in DMF for 3 h. Finally, resin was acetylated with Ac_2_O:DIEA:DMF (*v/v/v,* 1:1.2:3) for 45 min. Resin was washed with 3 × DMF, 3 × DCM, 1 × Et_2_O and dried in *vacuo* after finishing coupling and acetylation. During synthesis, the Fmoc group was removed with 2% piperidine and 2% DBU in DMF (3 + 17 min) and indicated by Kaiser test. The capacity of the resin was determined by UV–Vis measurement regarding to Fmoc chromophore amount (Fmoc-piperidine adduct) released by using 50% piperidine in DMF (Chan and White 2000). The final cleavage from S RAM Tentagel® resin was carried out with 50% TFA, 45% DCM, 2.5% TIS and 2.5% H_2_O mixture, or 95% TFA, 2.5% TIS and 2.5% H_2_O mixture (5–10 mL/g resin) for 3 h. Resin was washed with 2 × DMF, 3 × DCM and 2 × MeOH and solvent was removed in *vacuo*. The crude products were precipitated with diethyl ether.

### Zemplén deacetylation

The purified Ac-Gly-GlcAPC(Ac)-Gly-NH_2_ (**20 mg**) was dissolved in MeOH (2 ml) and 2 M NaOMe/MeOH (1 ml) solution was added. After 1 h at room temperature, the reaction was stopped with the addition of TFA (1 ml). The acidic solution was concentrated to get white solids (10 mg).

### Analytical data of chimera peptides

**Ac-Gly-GlcAPC(Ac)-Gly-NH**_**2**_

Rp-HPLC: 11.82 min, MS (ESI, *m/z*): [M + H]^+^ calcd. For 489.18; found: 489.15.

**Ac-Gly-GlcAPC(Ac)-GlcAPC(Ac)-Gly-NH**_**2**_

Rp-HPLC: 16.01 min, MS (ESI, *m/z*): [M + H]^+^ calcd. for 804.27; found: 804.21.

**Ac-Gly-GlcAPC-Gly-NH**_**2**_

Rp-HPLC: 3.61 min, MS (ESI, *m/z*): [M + H]^+^ calcd. for 363.05; found: 363.06.

### Supplementary Information

Below is the link to the electronic supplementary material.

Supplementary file1 (DOCX 1175 KB)

## Abbreviations

Boc-GlcAPC(Ac)-OH: 3,4,6-Tri-*O*-acetyl-2-deoxy-2-*tert*-butoxycarbonylamino-β-d-glucopyranosyl-1-carboxylic acid
DBU: 1,5-Diazabicyclo[5,4,0]undec-5-ene
DIC: *N,N*-Diisopropylcarbodiimide
DIEA: *N*,*N*-Diisopropylethylamine
Fmoc-GlcAPC(Ac)-OBt: 3,4,6-Tri-*O*-acetyl-2-deoxy-2-(9-fluorenylmethoxycarbonylamino)-β-d-glucopyranosyl-1-carboxylic acid HOBt ester
Fmoc-GlcAPC(Ac)-OH: 3,4,6-Tri-*O*-acetyl-2-deoxy-2-(9-fluorenylmethoxycarbonylamino)-β-d-glucopyranosyl-1-carboxylic acid
Fmoc-GlcAPC(Bn)-OH: 3,4,6-Tri-*O*-benzyl-2-deoxy-2-(9-fluorenylmethoxycarbonylamino)-β-d-glucopyranosyl-1-carboxylic acid
Fmoc-GlcAPC-OBt: 2-Deoxy-2-(9-fluorenylmethoxycarbonylamino)-β-d-glucopyranosyl-1-carboxylic acid HOBt ester
Fmoc-GlcAPC-OH: 2-Deoxy-2-(9-fluorenylmethoxycarbonylamino)-β-d-glucopyranosyl-1-carboxylic acid
Fmoc-β-SAA-OBt: Fmoc protected β-sugar amino acid HOBt ester
H-GlcAPC-OH: 2-Amino-2-deoxy-β-d-glucopyranosyl-1-carboxylic acid
H-Gly-OMe: Glycine methyl ester
HOBt: Hydroxybenzotriazole
*N*-Fmoc: *N*-9-Fluorenylmethoxycarbonyl
*N*-Phth: *N*-Phtaloyl group
OAc: *O*-Acetyl group
Phth-GlcAPC(Ac)-NH_2_: 3,4,6-Tri-*O*-acetyl-2-deoxy-2-phthalimido-β-d-glucopyranosyl-1-carboxamide
PyBOP: Benzotriazol-1-yloxytripyrrolidinophosphonium hexafluorophosphate
d-Rib: D-Ribose
-RibAFU(ip)-: 3-Amino-3-deoxy-1,2-di-*O*-isopropylidene-α-d-ribofuranuronic acid
rt: Room temperature
SPPS: Solid-phase peptide synthesis
TMSCN: Trimethylsilyl cyanide
d-Xyl: D-Xylose
-XylAFU(ip)-: 3-Amino-3-deoxy-1,2-di-*O*-isopropylidene-α-d-xylofuranuronic acid
β-SAA: β-Sugar amino acid
