# Supplementary material for: Acetyl group for proper protection of β-sugar-amino acids used in SPPS
Source: Amino Acids. 2023 Jun 21;55(8):969–79. doi: 10.1007/s00726-023-03278-1 (PMC10514111; doi:10.1007/s00726-023-03278-1)
Supplement: Supplementary file 1 — Supplementary file1 (DOCX 1175 KB) [file 726_2023_3278_MOESM1_ESM.docx]

**SUPPLEMENTARY INFORMATION**

**Acetyl group for proper protection of** **β-sugar-amino acids used in SPPS**

István Varga^a,b^, Viktória Goldschmidt Gőz^c^, István Pintér,^a^ Antal Csámpai^d^ and András Perczel^a,c^, ⃰

^a^ Laboratory of Structural Chemistry and Biology, Institute of Chemistry, Eötvös Loránd University, Pázmány P. stny. 1/A, 1117 Budapest, Hungary

^b^ György Hevesy Doctoral School of Chemistry, Eötvös University, Budapest, Hungary

^c^ MTA-ELTE Protein Modeling Research Group, Pázmány P. stny. 1/A, 1117 Budapest, Hungary
^d^ Organic Chemistry Department, Eötvös Loránd University, Pázmány P. stny. 1/A, 1117 Budapest, Hungary

⃰ corresponding author: perczel.andras@ttk.elte.hu

**Table of content**

[^1^H-NMR Spectra of the active esters formation and coupling reactions 2](#_Toc125657112)

[HPLC chromatograms 7](#_Toc125657113)

[MS spectra 10](#_Toc125657114)

# **^1^H-NMR Spectra of the active esters formation and coupling reactions**

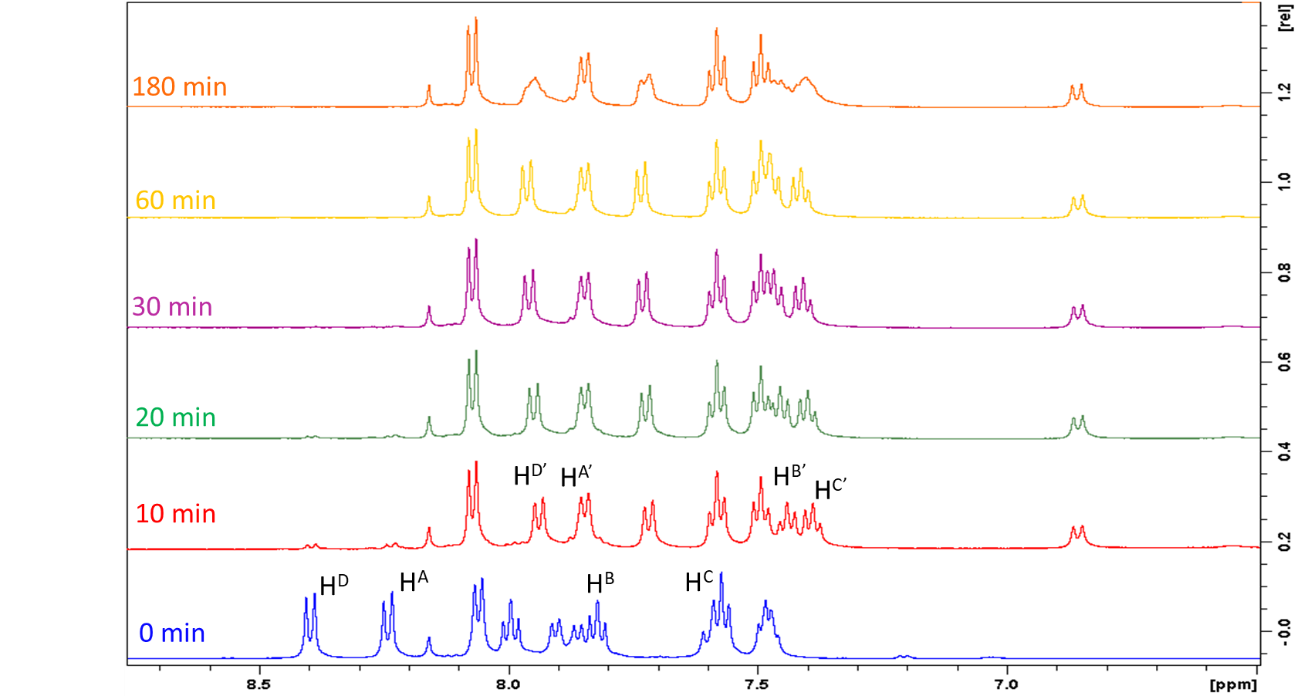


**SFigure 1.** Active ester formation of Fmoc-GlcAPC-OH (**1**) with PyBOP/DIEA, 500 MHz, DMF-d_7_ as function of the time (0 ≤ *t*(min) ≤180)

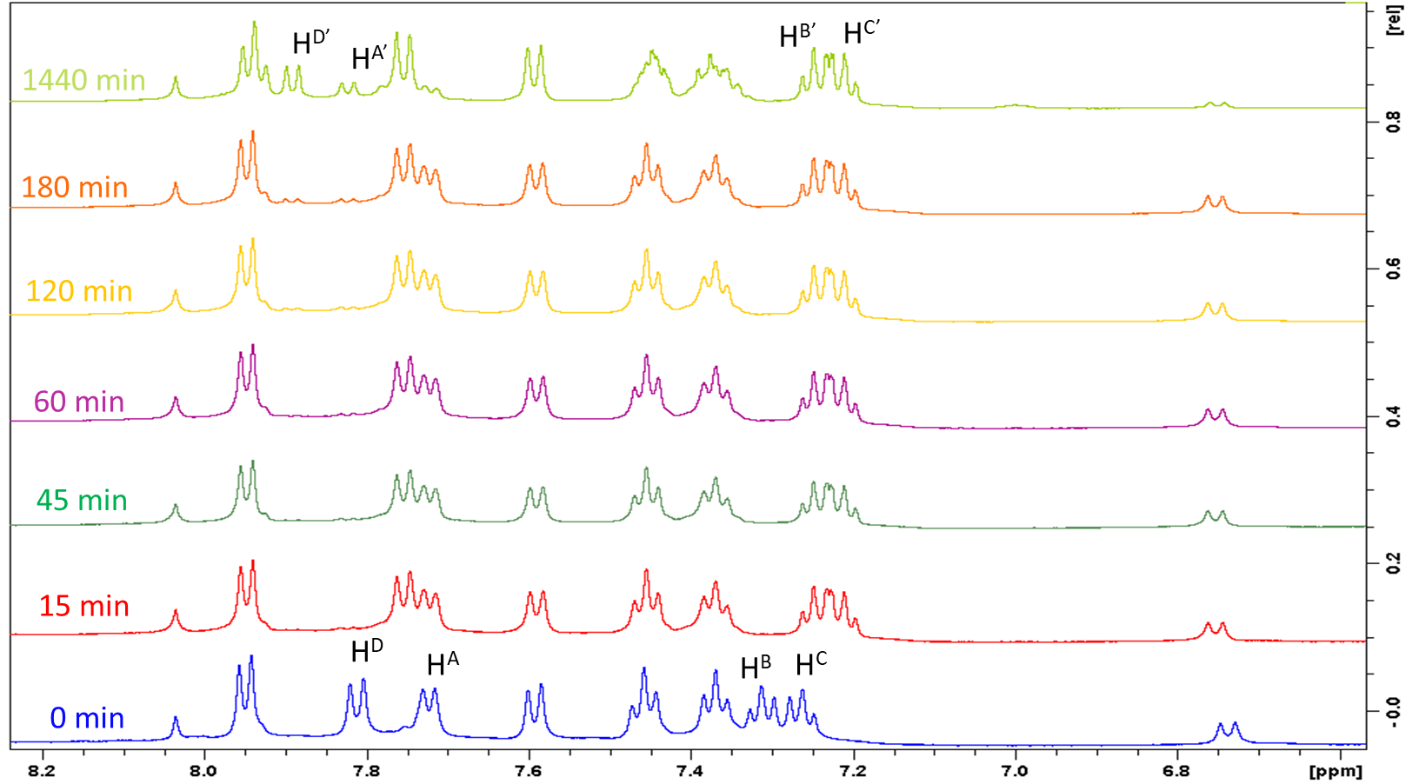


**SFigure 2.** Coupling of Fmoc-GlcAPC-OBt (**11**) with H-Gly-OMe, 500 MHz, in DMF-d_7_ as function of the time (0 ≤ *t*(min) ≤ 1440)


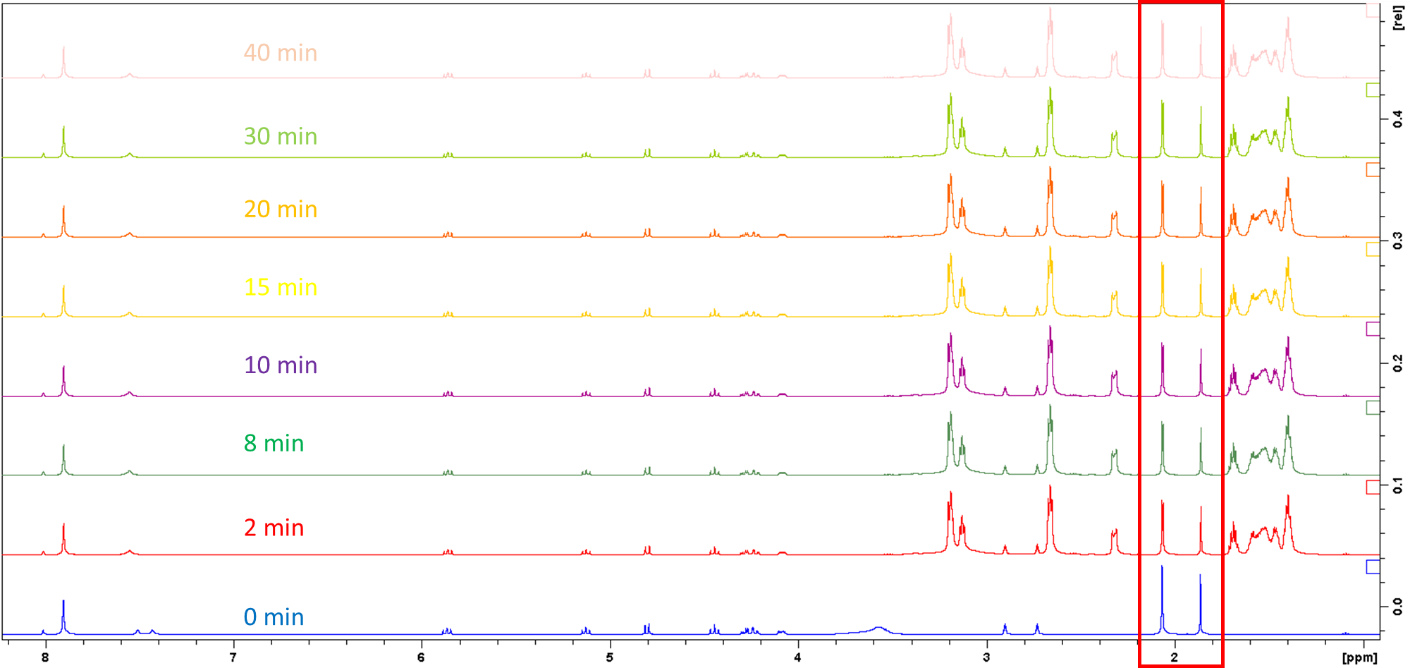


**SFigure 3.** Acetyl group stability of Phth-GlcAPC(Ac)-NH_2_ (**13**) in 2% piperidine and 2% DBU in DMF-d_7_ as function of the time (0 ≤ *t*(min) ≤ 40)


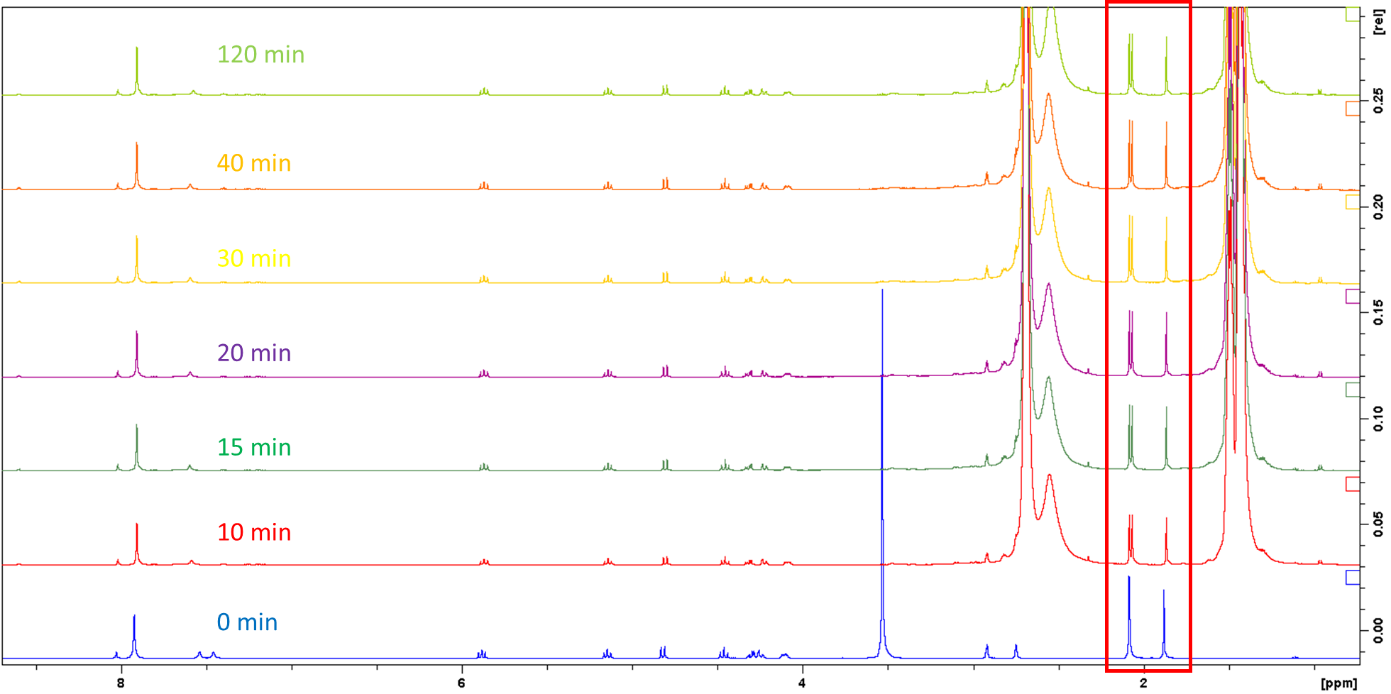


**SFigure 4.** Acetyl group stability of Phth-GlcAPC(Ac)-NH_2_ (**13**) in 20% piperidine in DMF-d_7_ as function of the time (0 ≤ t(min) ≤120)


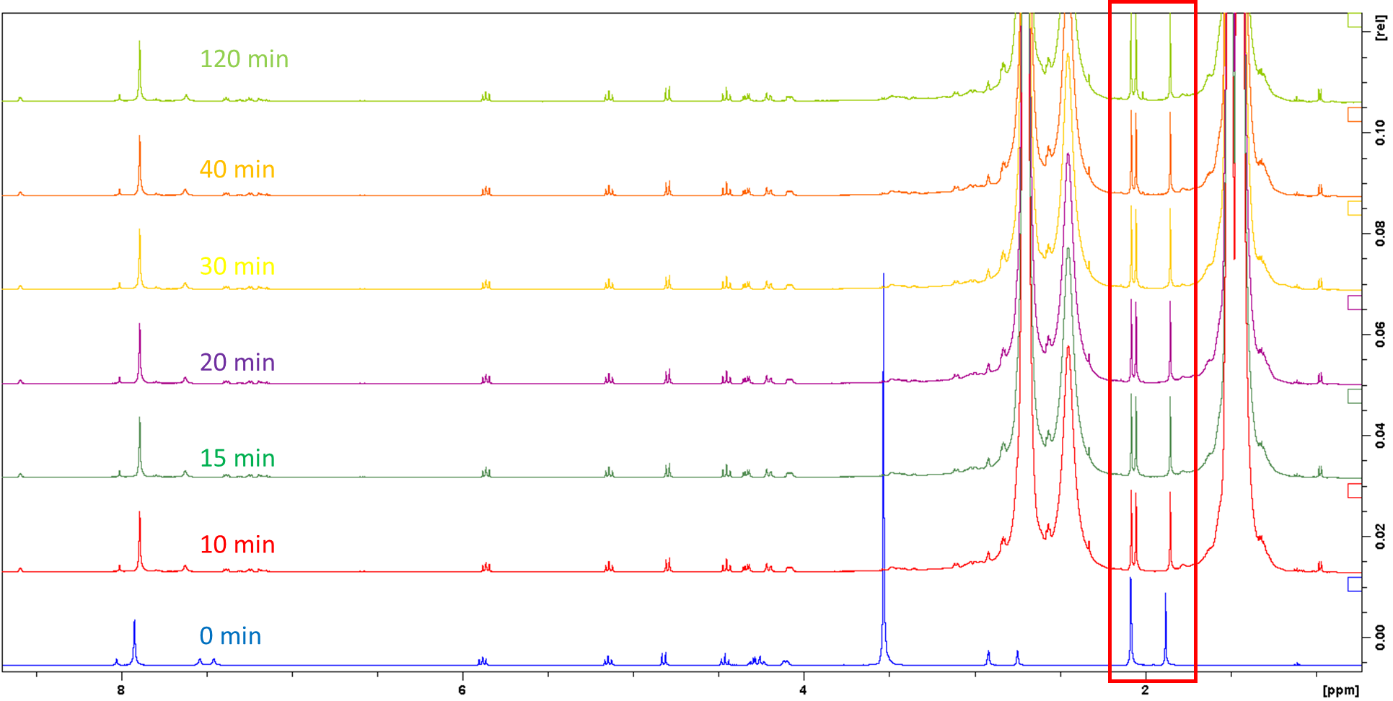


**SFigure 5**. Acetyl group stability of Phth-GlcAPC(Ac)-NH_2_ (**13**) in 40% piperidine in DMF-d_7_ as function of the time (0 ≤ *t*(min) ≤120)


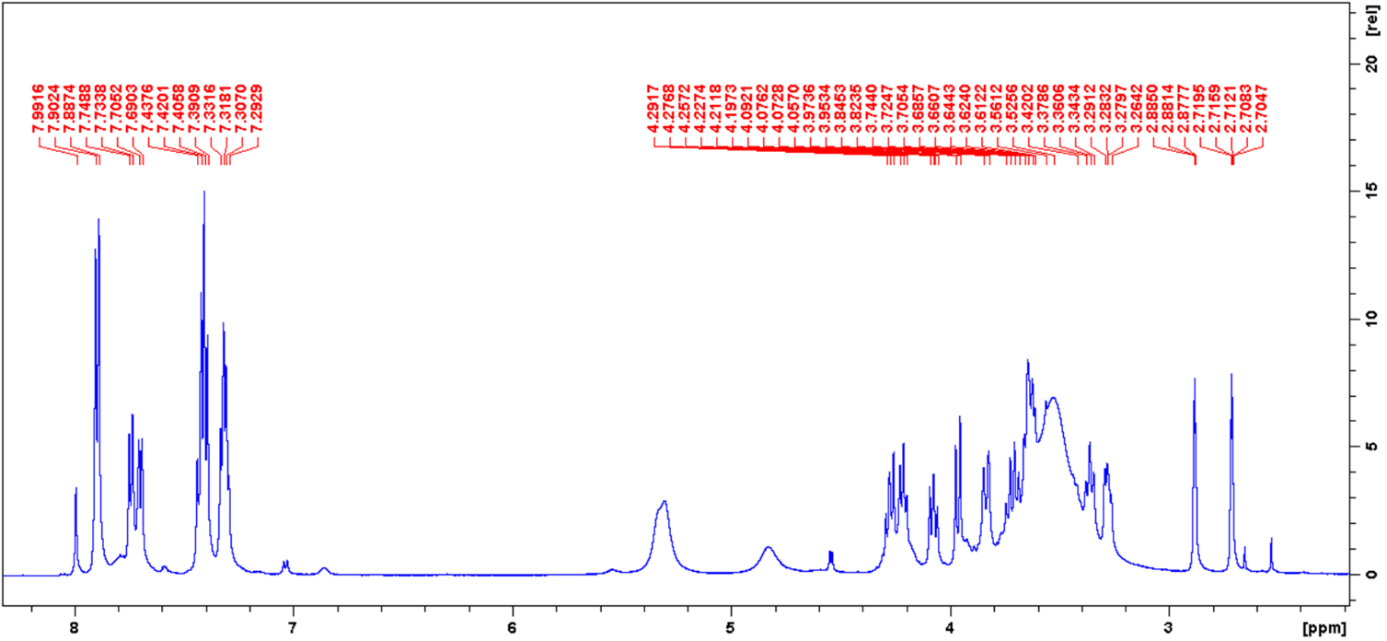


**SFigure 6.** ^1^H NMR spectrum of Fmoc-GlcAPC-OH (**1**), at 500 MHz, in DMF-d_7_


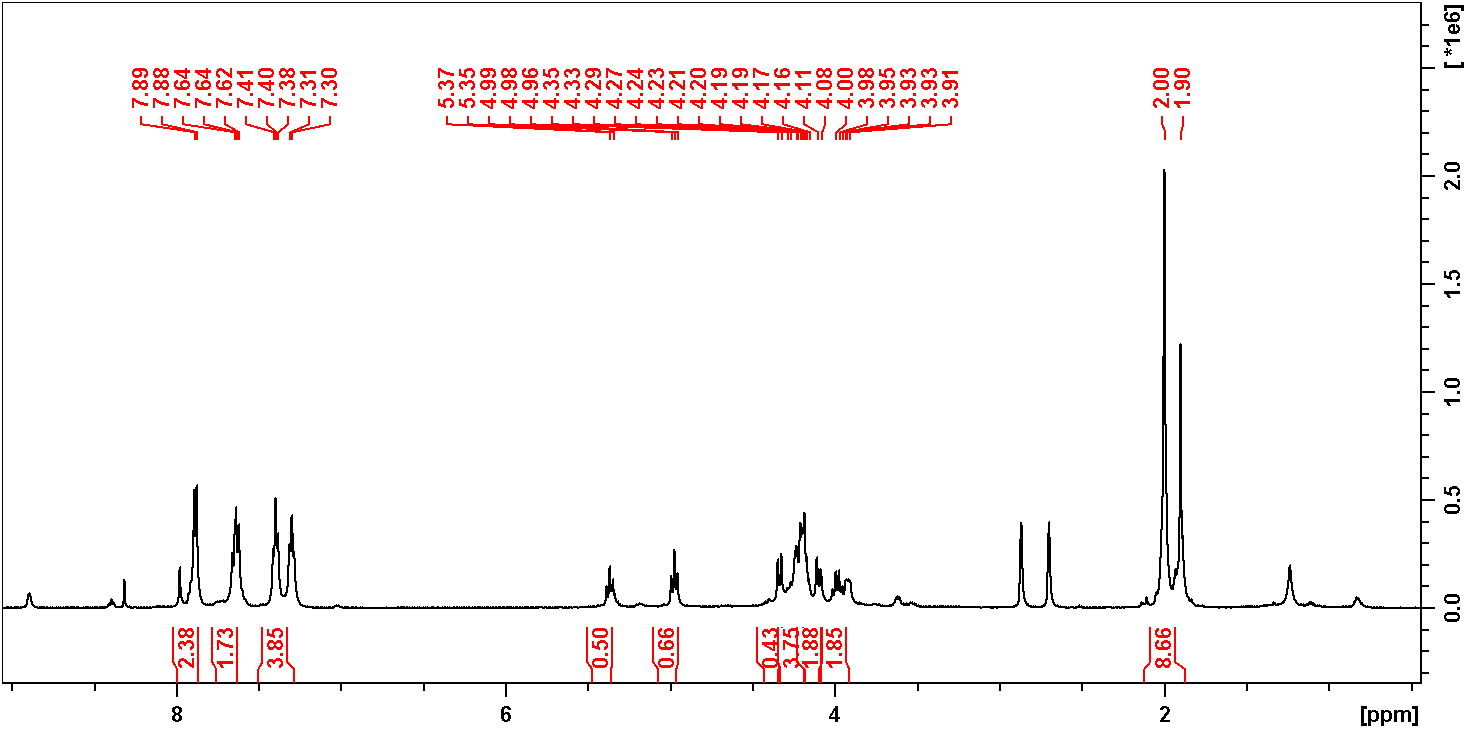


**SFigure 7.** ^1^H NMR spectrum of Fmoc-GlcAPC(Ac)-OH (**2**), at 500 MHz, in DMF-d_7_


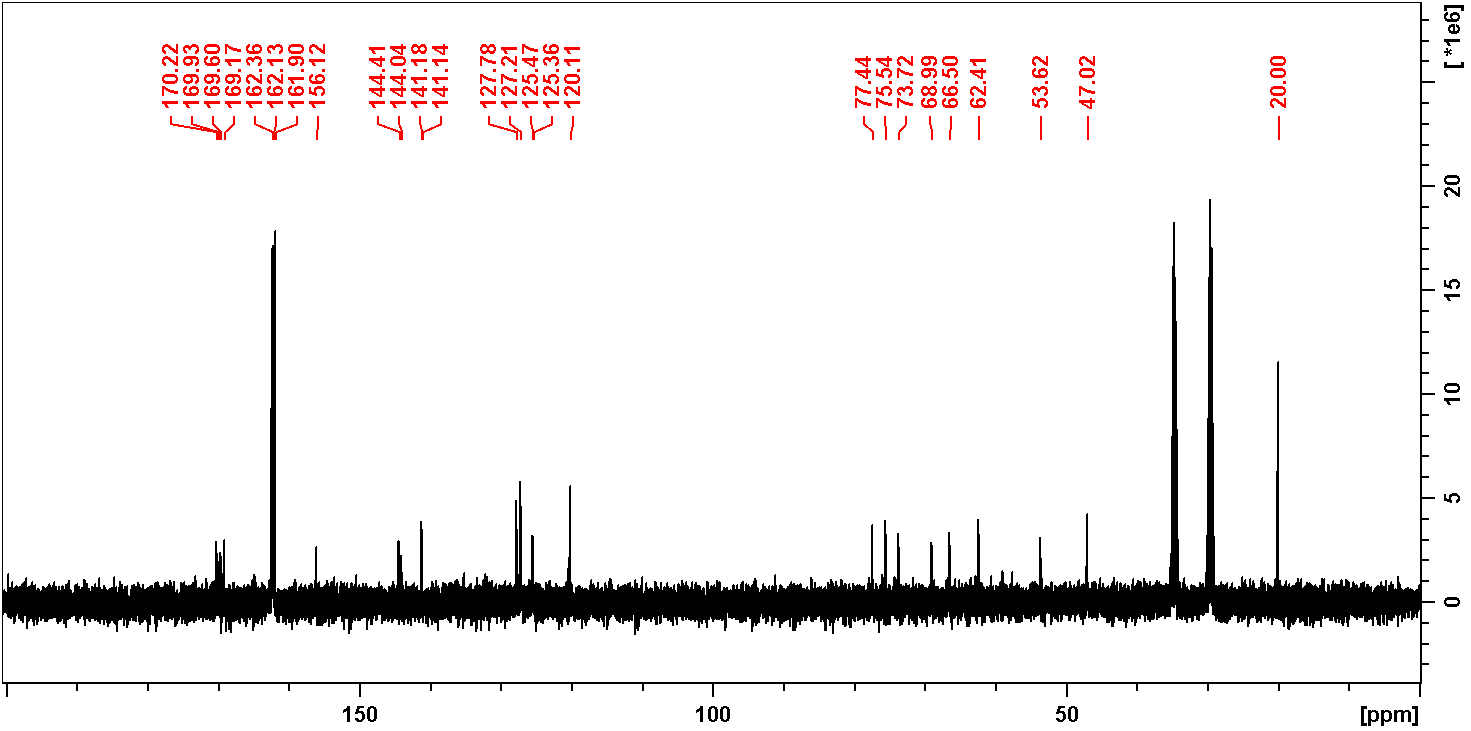


**SFigure 8.** ^13^C NMR spectrum of Fmoc-GlcAPC(Ac)-OH (**2**), at 500 MHz, in DMF-d_7_

# **HPLC chromatograms**


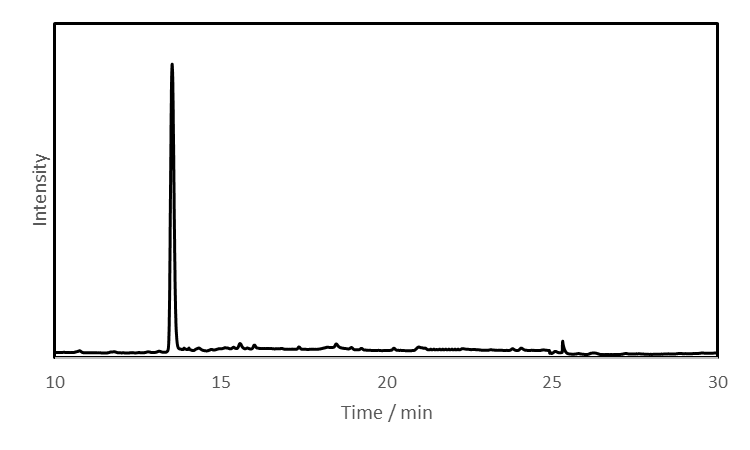

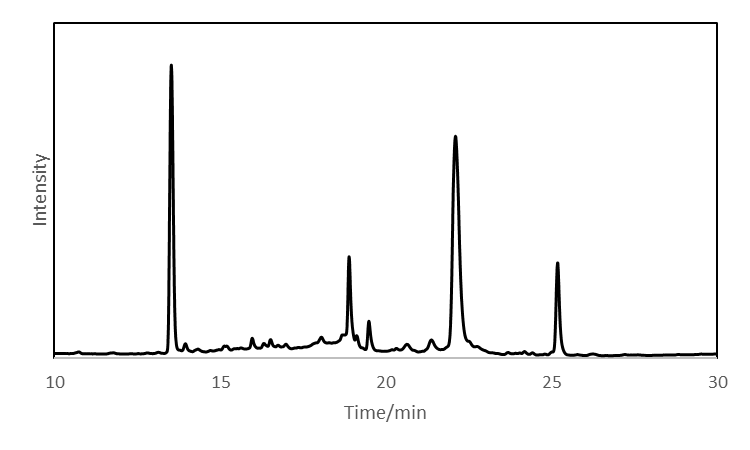


**purified**

**crude**

**SFigure 9.** RP-HPLC chromatogram of the crude and purified Ac-GXG-NH_2_, where X=-GlcAPC(Ac)- moiety

**SFigure 10.** RP-HPLC chromatogram of the crude Ac-GXG-NH_2_, where X=-GlcAPC(Ac)- moiety using 40°C end cleavage method


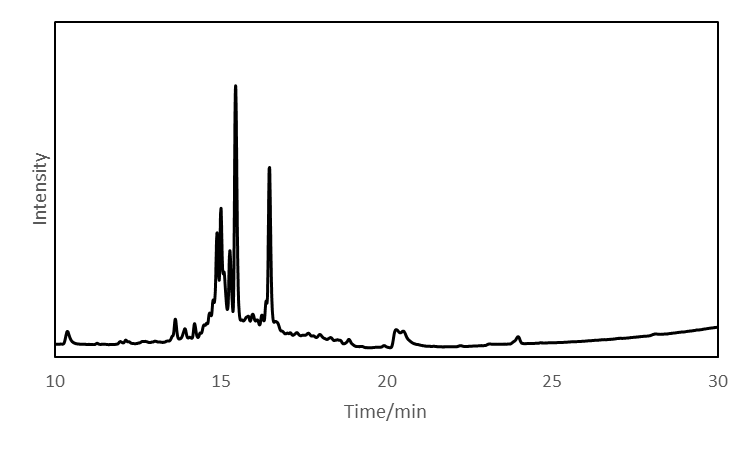


**SFigure 11**. RP-HPLC chromatogram of the crude Ac-GXXG-NH_2_, where X=-GlcAPC- moiety


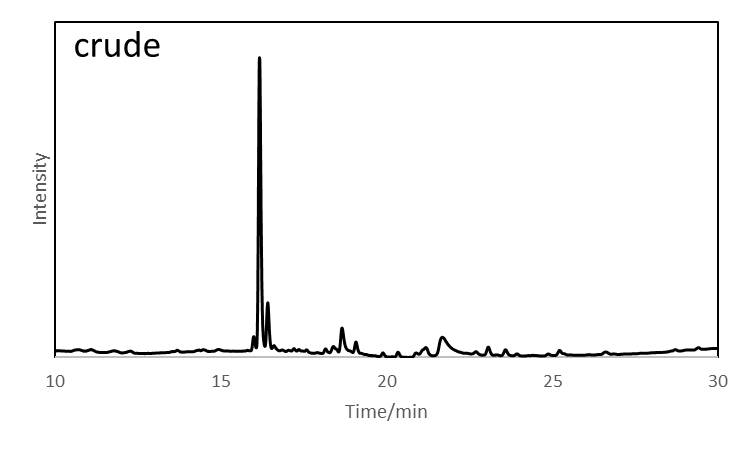

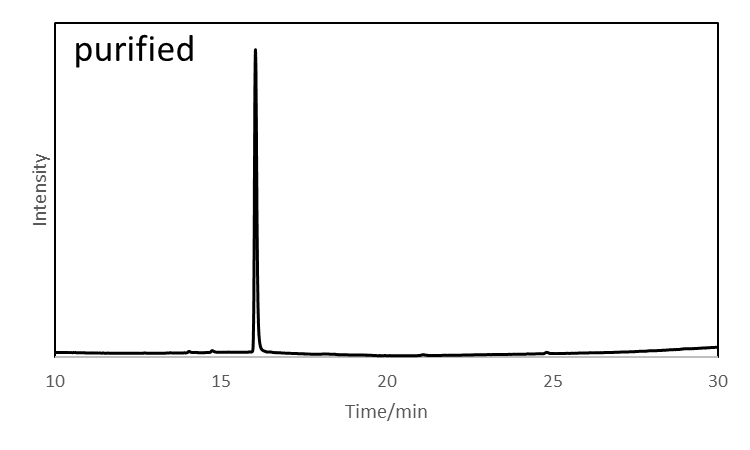


**SFigure 12.** RP-HPLC chromatogram of the crude and purified Ac-GXXG-NH_2_, where X=-GlcAPC(Ac)- moiety


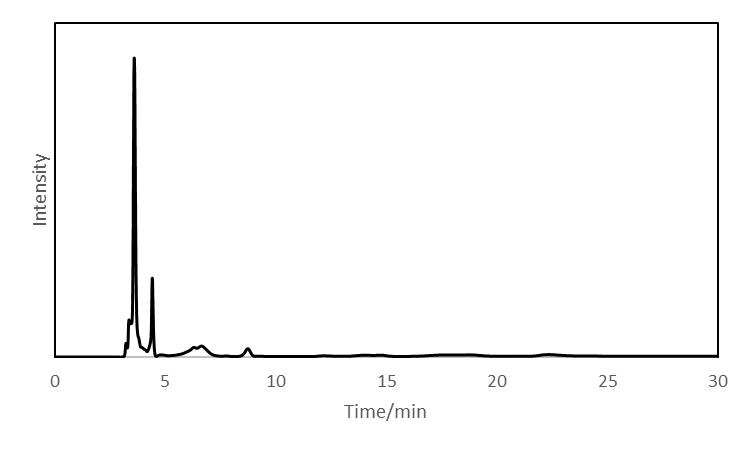


**SFigure 13.** Zemplén deacetylation: RP-HPLC chromatogram of the crude Ac-GXG-NH_2_, where
X=-GlcAPC- moiety

# **MS spectra**


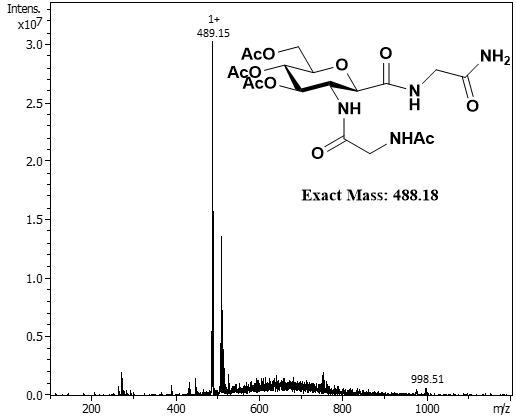


**SFigure 14**. MS spectrum of the crude Ac-GXG-NH_2_, where X=-GlcAPC(Ac)- moiety (coupling with Fmoc-GlcAPC(Ac)-OH, **2**, β-SAA, and cleaved with 95% TFA at 40°C)


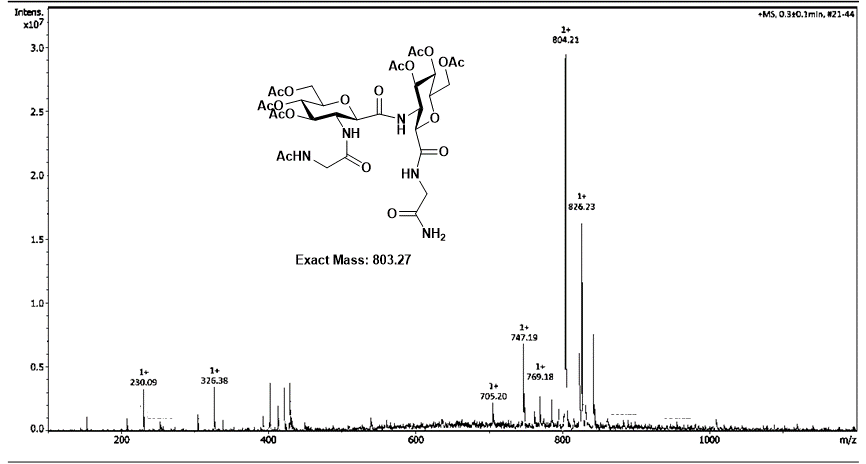


**SFigure 15.** MS spectrum of the crude Ac-GXXG-NH_2_, where X=-GlcAPC(Ac)- moiety using 95 % TFA final cleavage (coupling with Fmoc-GlcAPC(Ac)-OH, **2**, β-SAA)


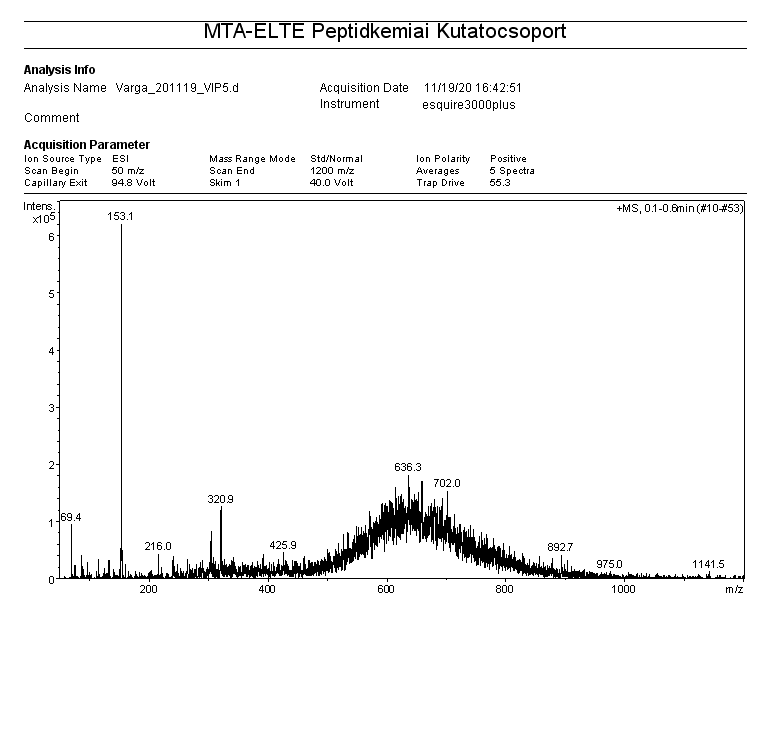


**SFigure 16.** MS spectrum of the crude Ac-GXXG-NH_2_, where X=-GlcAPC- moiety (coupling with Fmoc-GlcAPC-OH, **1** β-SAA)


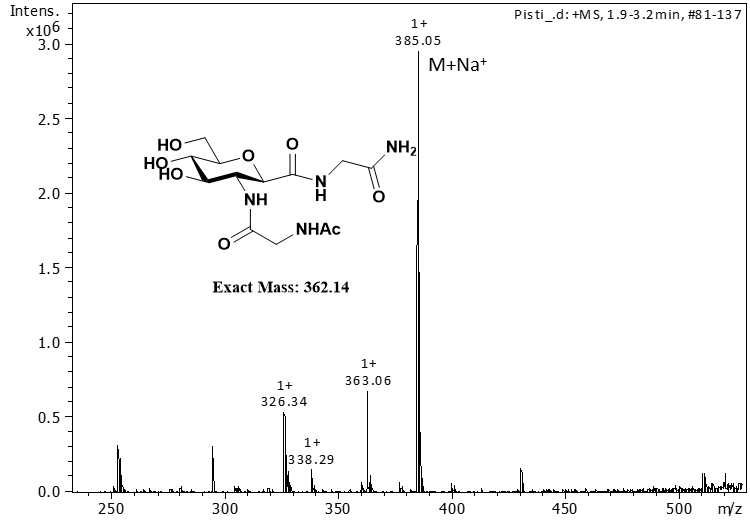


**SFigure 17.** Zemplén deacetylation: MS spectrum of the crude Ac-GXG-NH_2_, where X=-GlcAPC- moiety
